# Supplementary figures and images for: PP6 regulation of Aurora A–TPX2 limits NDC80 phosphorylation and mitotic spindle size
Source: J Cell Biol. 2023 Mar 10;222(5):e202205117. doi: 10.1083/jcb.202205117 (PMC10041653; doi:10.1083/jcb.202205117)

**Fig. 3C**

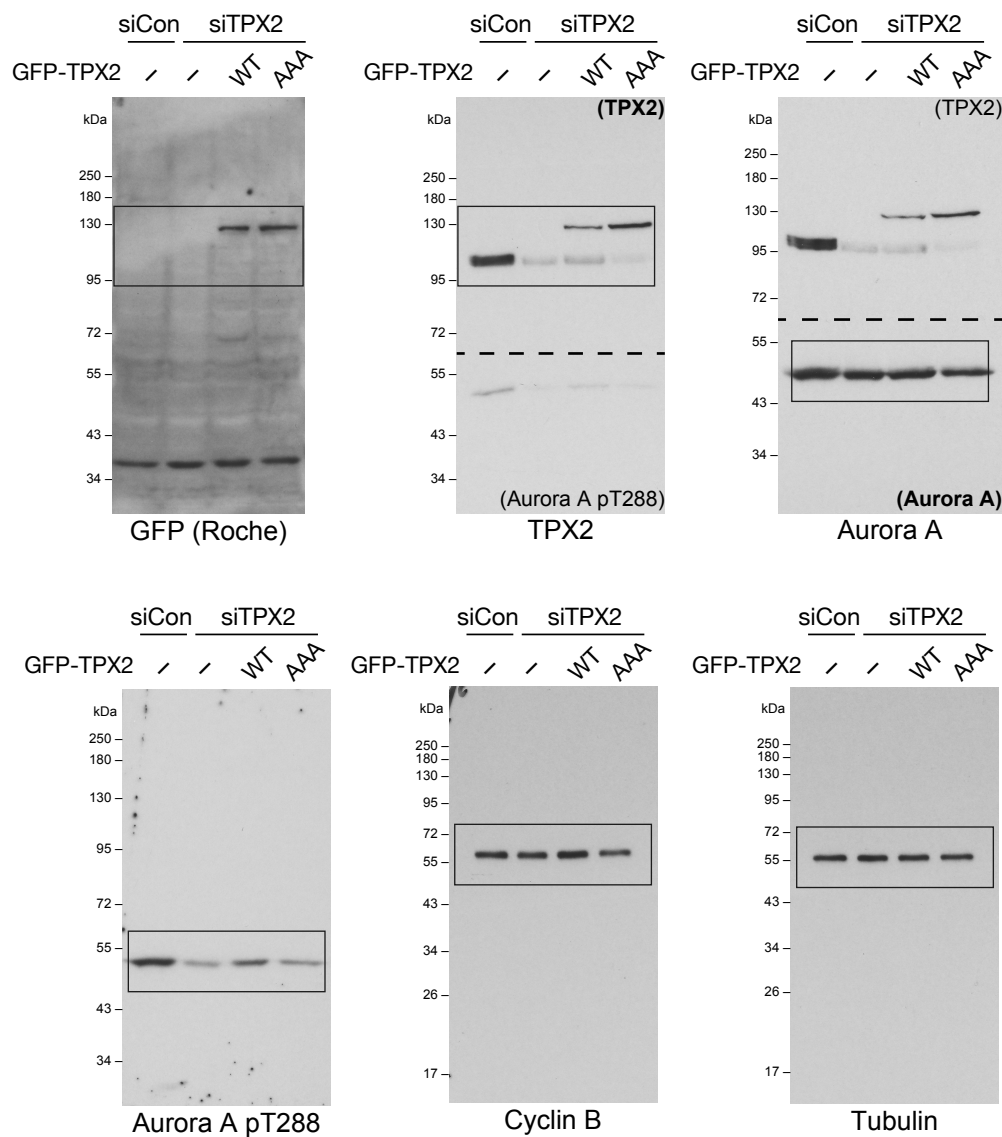

ECL-Film exposures

Supplement: SourceData F3 — contains original blots for Fig. 3. [file JCB_202205117_SourceDataF3.pdf]

**Fig. 6B (Parental)**

**Fig. 6B (PPP6C KO)**

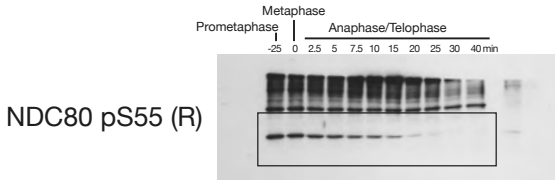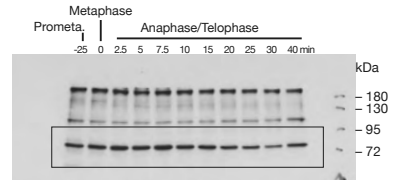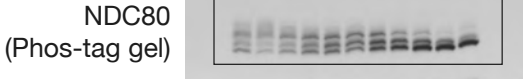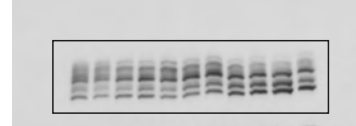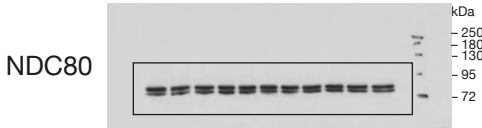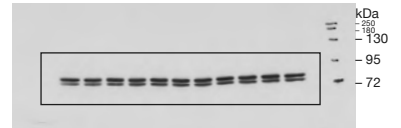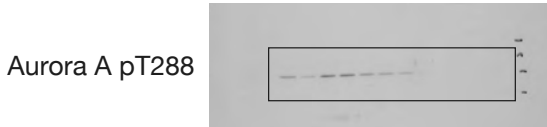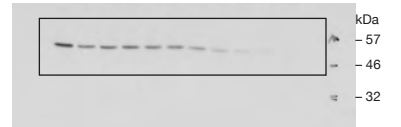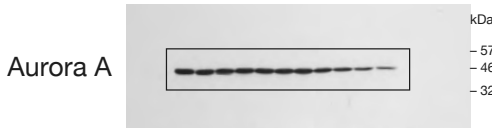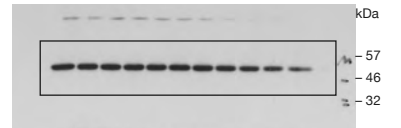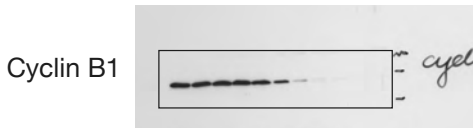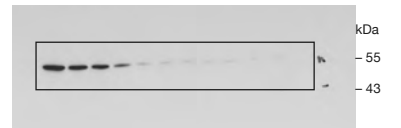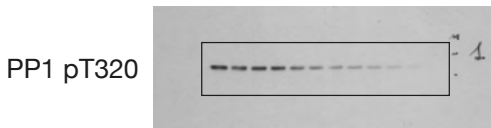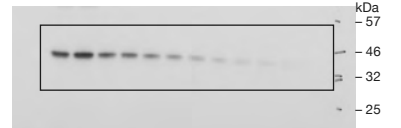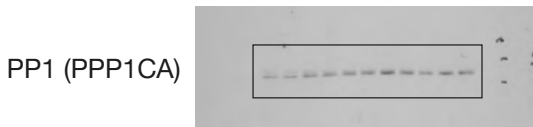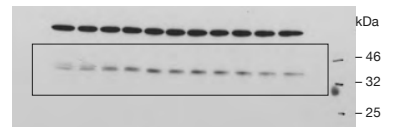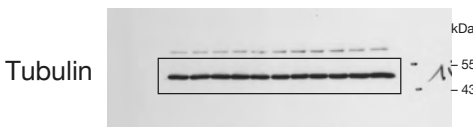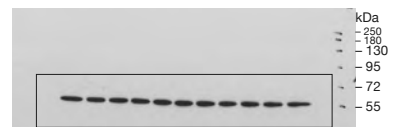

ECL-Film exposures

Supplement: SourceData F6 — contains original blots for Fig. 6. [file JCB_202205117_SourceDataF6.pdf]

**Fig. 9D**

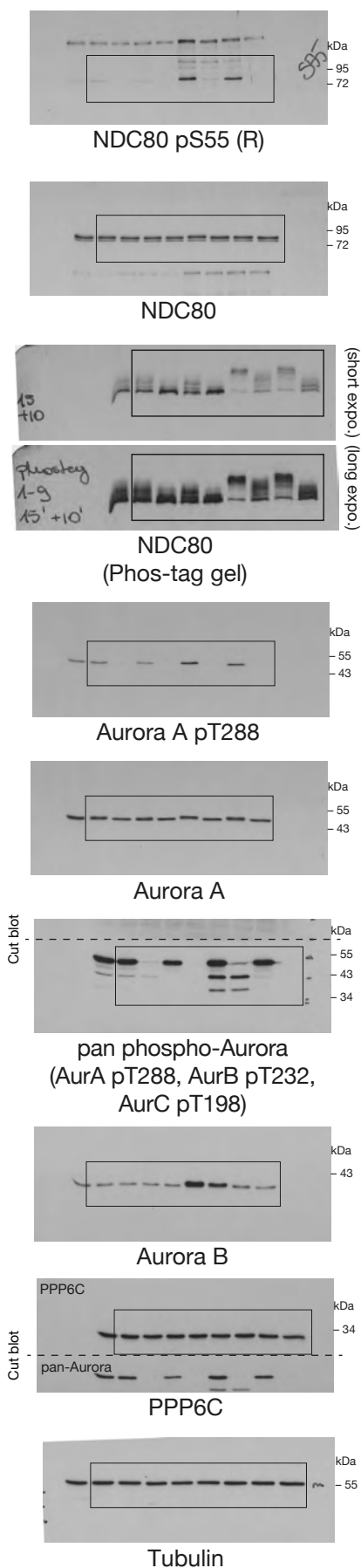

**Fig. 9E**

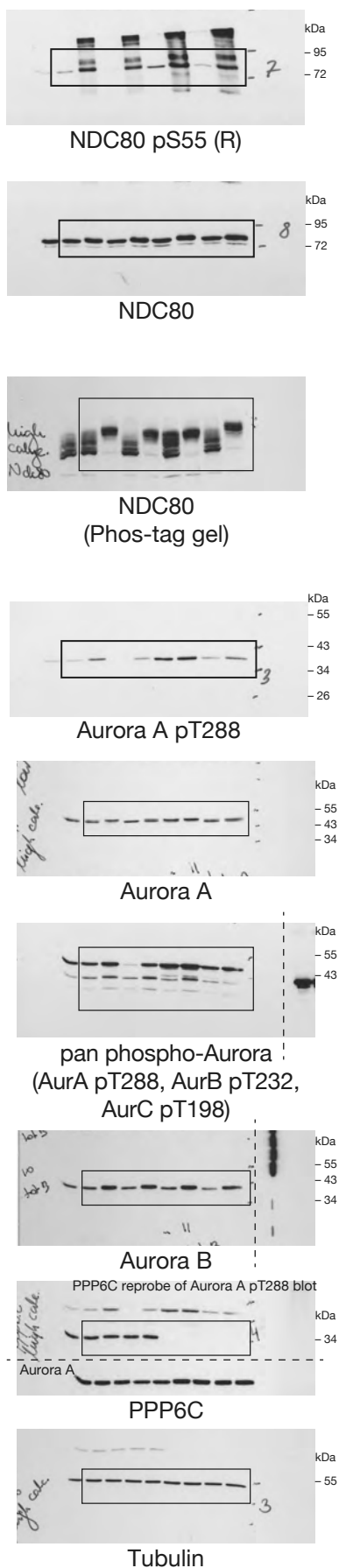

Supplement: SourceData F9 — contains original blots for Fig. 9. [file JCB_202205117_SourceDataF9.pdf]

**Fig. S1B**

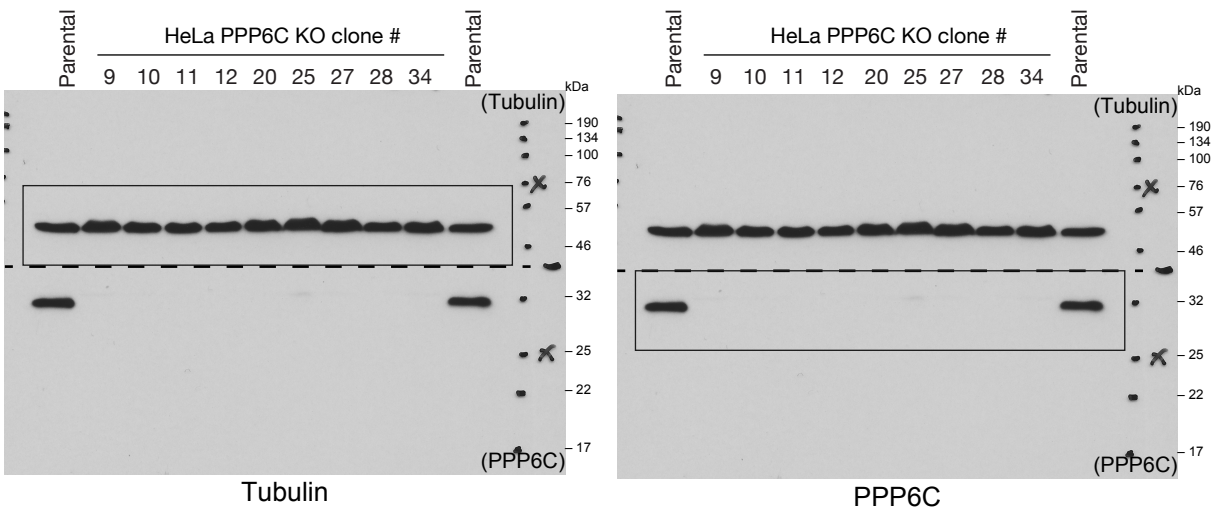

**Fig. S1C**

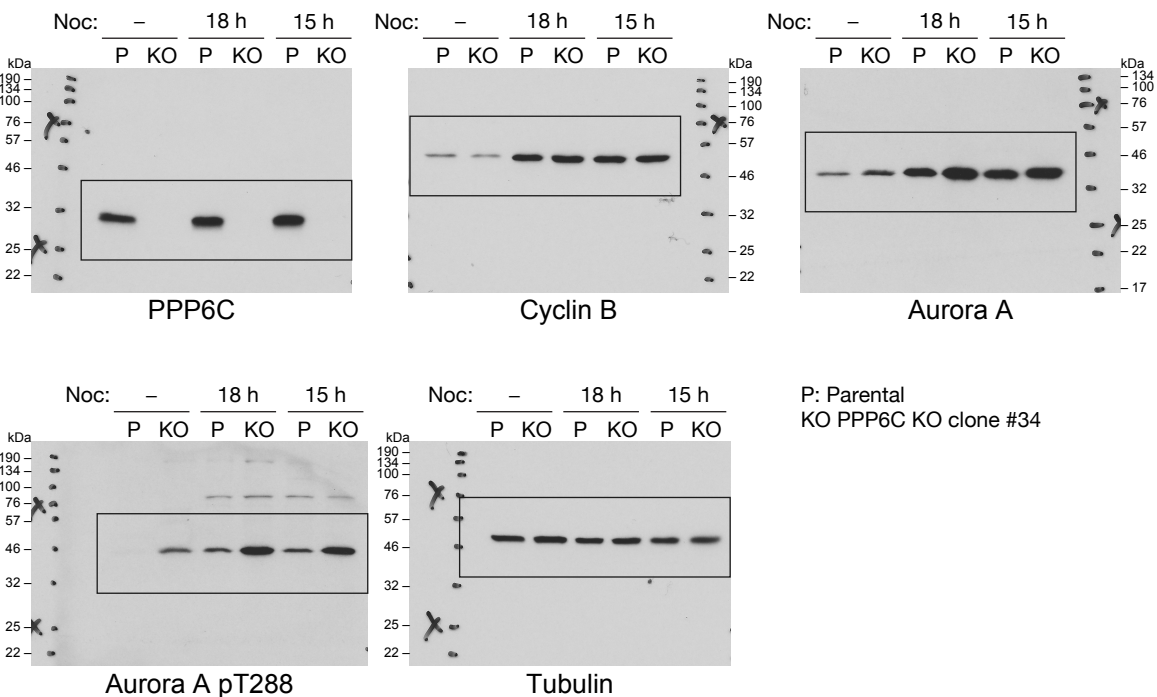

Supplement: SourceData FS1 — contains original blots for Fig. S1. [file JCB_202205117_SourceDataFS1.pdf]

**Fig. S2C**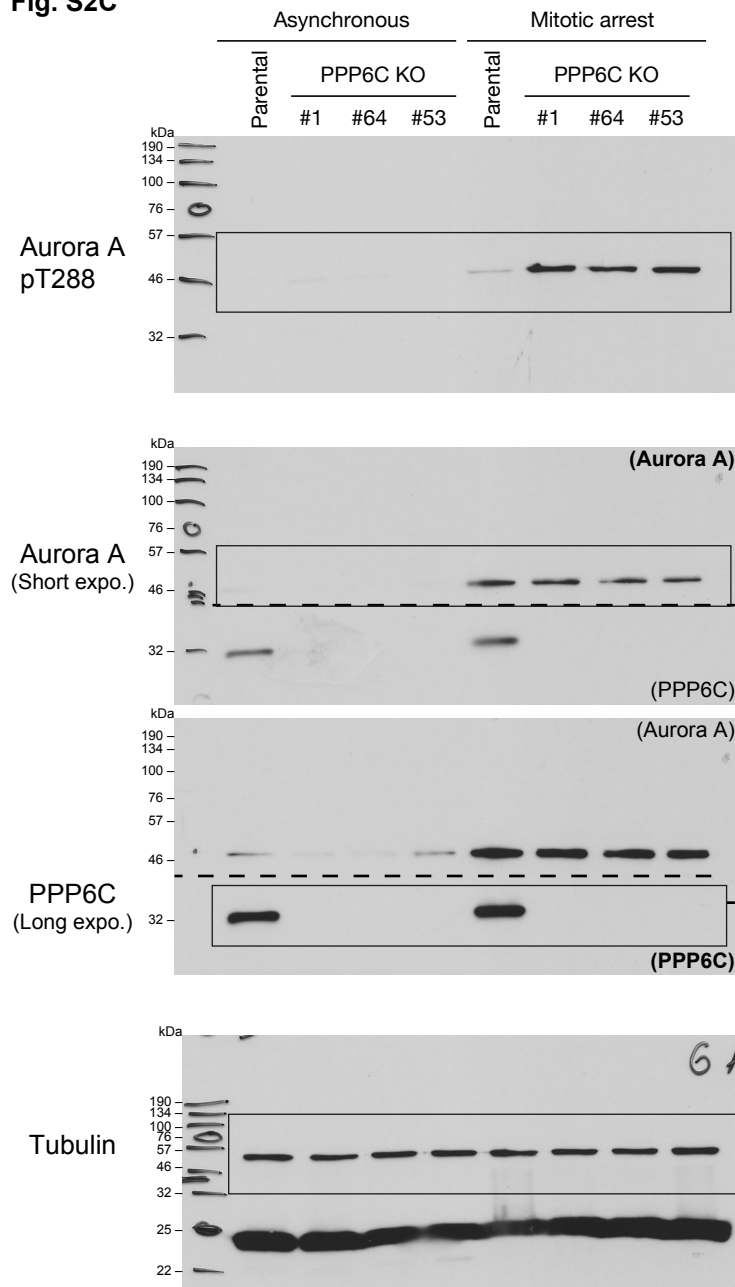**Fig. S2I**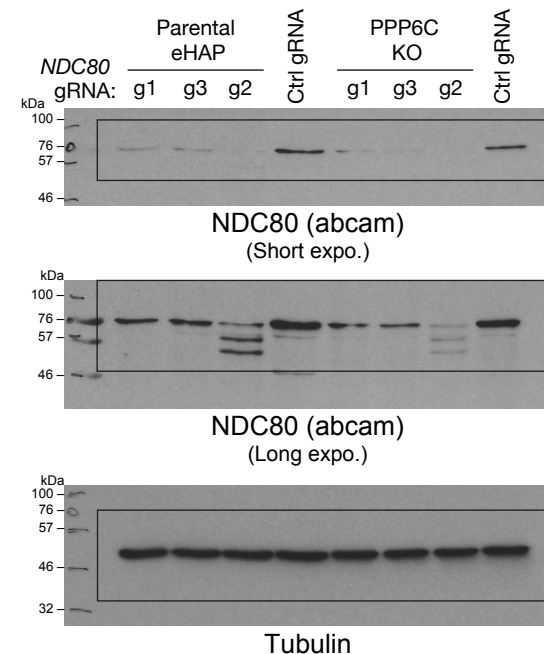

ECL-Film exposures

Supplement: SourceData FS2 — contains original blots for Fig. S2. [file JCB_202205117_SourceDataFS2.pdf]

Fig. S3B

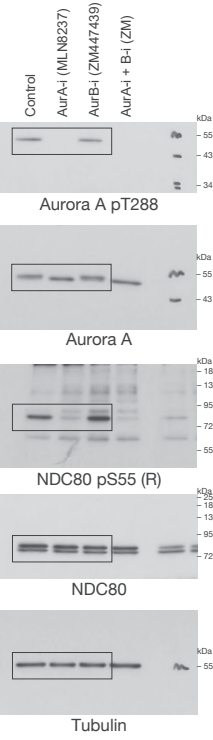

Fig. S3D extended analysis from Fig. 4D

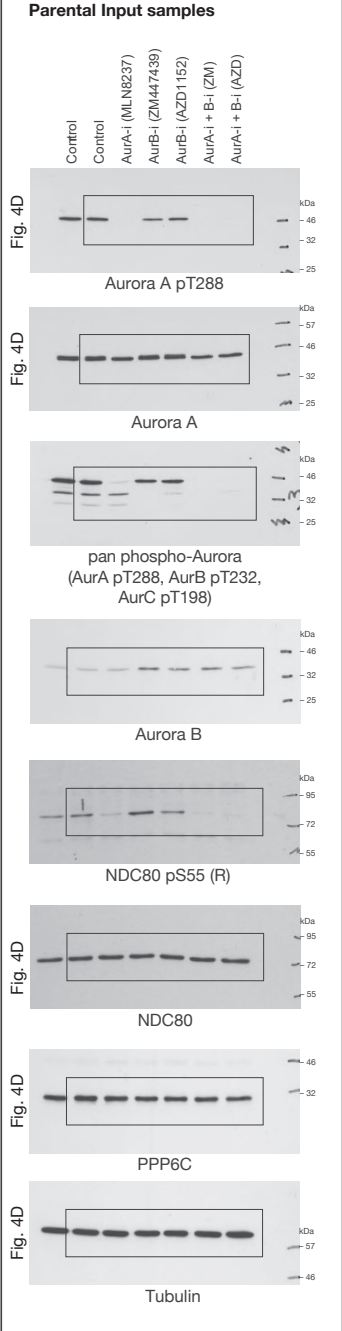

Parental NDC80 IP samples

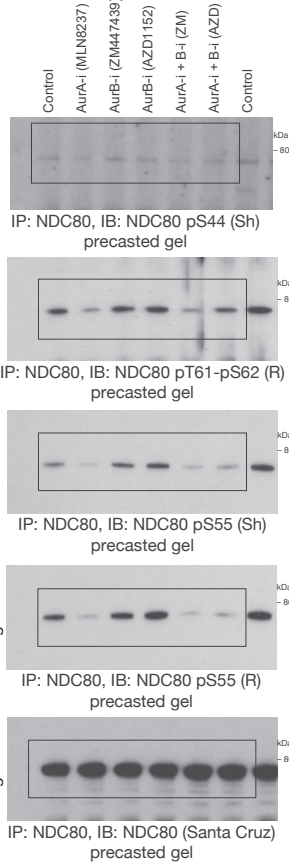

Fig. S3E

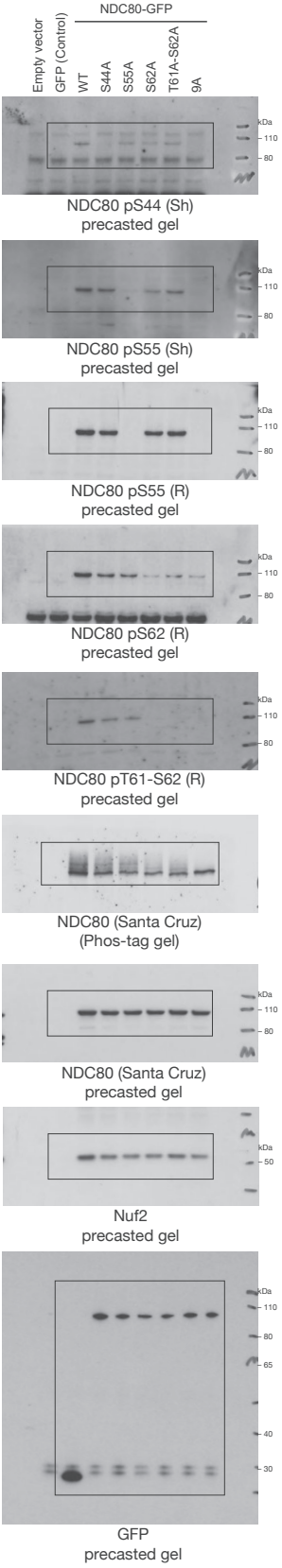

Supplement: SourceData FS3 — contains original blots for Fig. S3. [file JCB_202205117_SourceDataFS3.pdf]

**Fig. S4C**

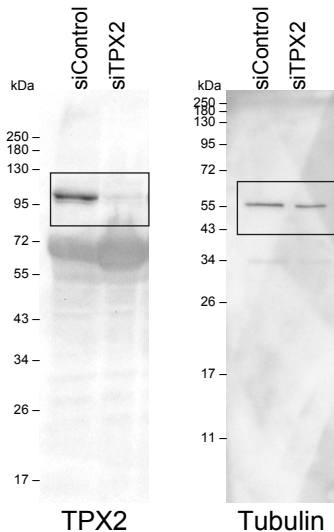

ECL with Bio-Rad ChemiDoc

Supplement: SourceData FS4 — contains original blots for Fig. S4. [file JCB_202205117_SourceDataFS4.pdf]
