# Supplementary material for: PP6 regulation of Aurora A–TPX2 limits NDC80 phosphorylation and mitotic spindle size
Source: J Cell Biol. 2023 Mar 10;222(5):e202205117. doi: 10.1083/jcb.202205117 (PMC10041653; doi:10.1083/jcb.202205117)

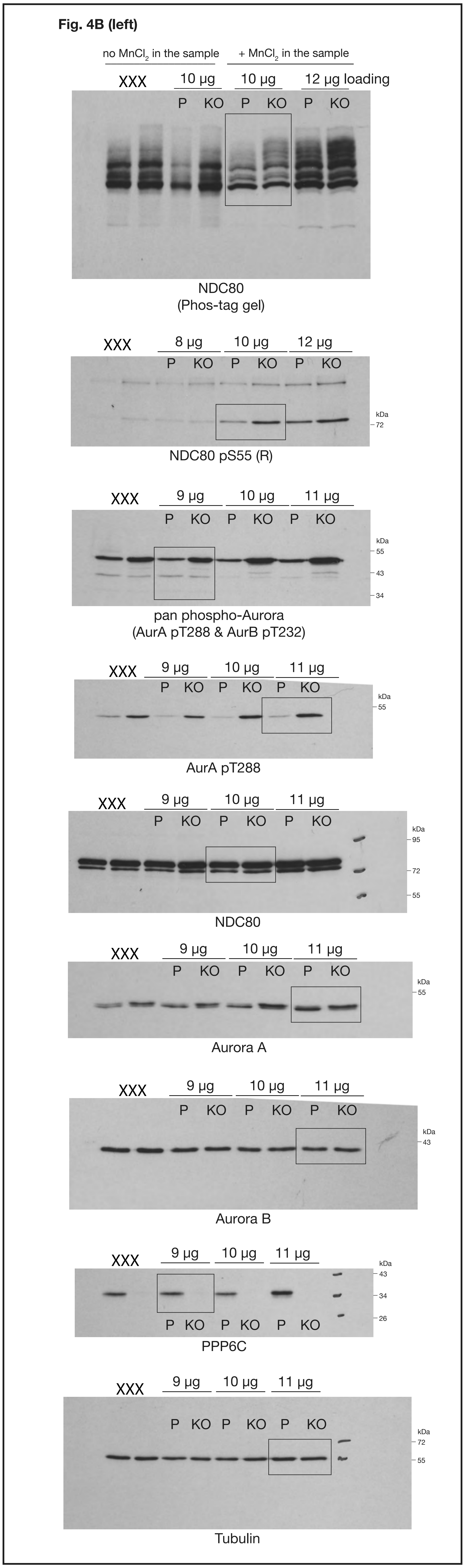

P: Parental
KO: PPP6C KO

ECL-Film exposures

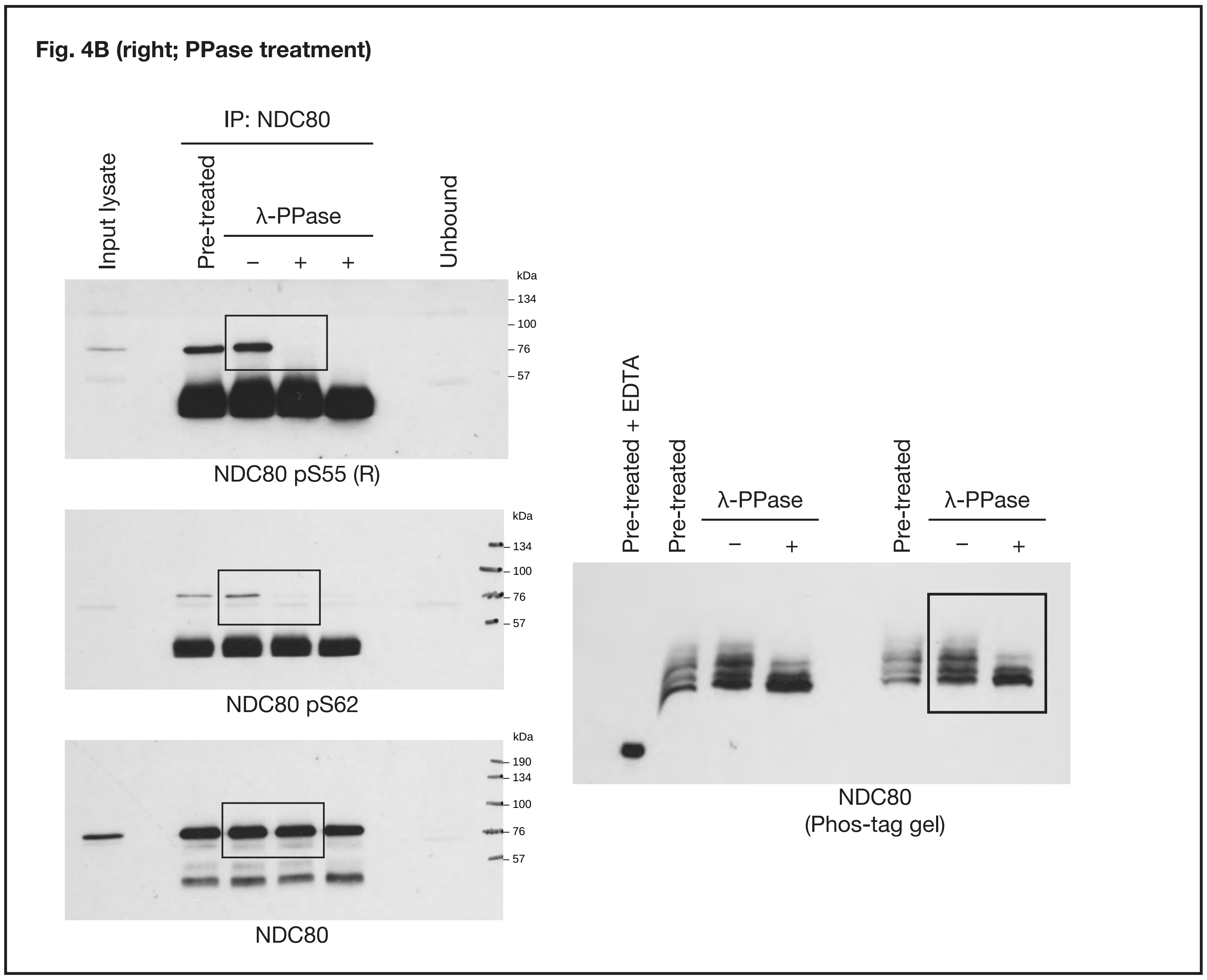

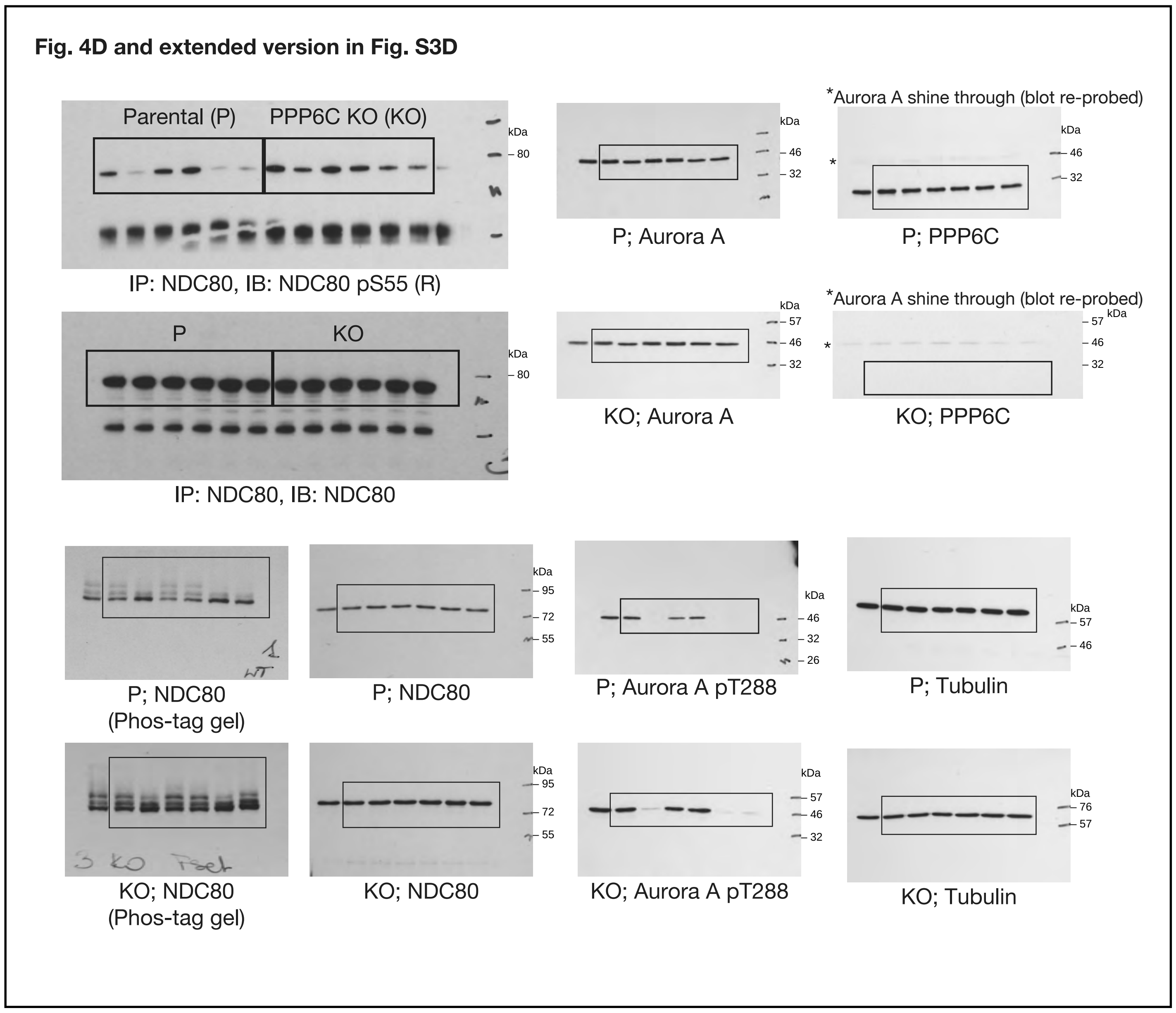

Supplement: SourceData F4 — contains original blots for Fig. 4. [file JCB_202205117_SourceDataF4.pdf]
