# Supplementary material for: PP6 regulation of Aurora A–TPX2 limits NDC80 phosphorylation and mitotic spindle size
Source: J Cell Biol. 2023 Mar 10;222(5):e202205117. doi: 10.1083/jcb.202205117 (PMC10041653; doi:10.1083/jcb.202205117)
Supplement: SourceData F5 — contains original blots for Fig. 5. [file JCB_202205117_SourceDataF5.pdf]

Fig. 5A (Aurora B inhibitor time course)

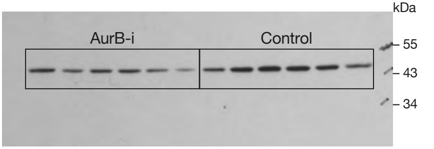

Aurora A pT288

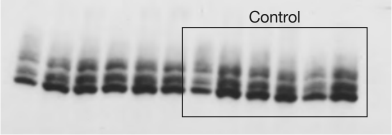

NDC80
(Phos-tag gel)

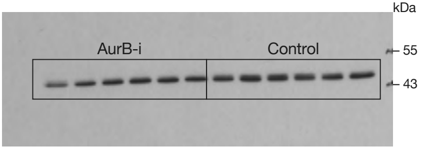

Aurora A pT288

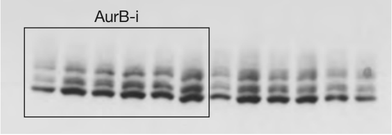

NDC80
(Phos-tag gel)

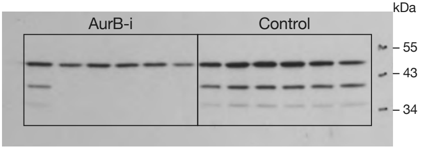

pan phospho-Aurora
(AurA pT288, AurB pT232,
AurC pT198)

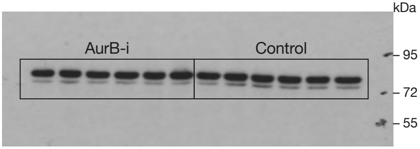

NDC80

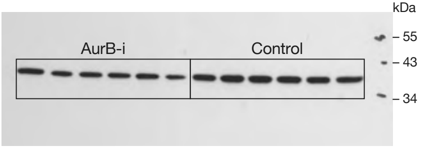

Aurora B

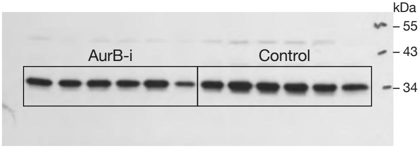

PPP6C

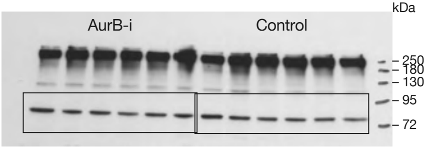

NDC80 pS55 (R)

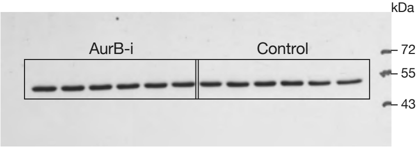

Tubulin

ECL-Film exposures
